# Wage Growth, Landholding, and Mechanization in Chinese Agriculture

**DOI:** 10.1016/j.worlddev.2016.05.002

**Published:** 2016-10

**Authors:** Xiaobing Wang, Futoshi Yamauchi, Keijiro Otsuka, Jikun Huang

**Affiliations:** a Peking University and Center for Chinese Agricultural Policy, China; b World Bank, Washington DC, USA; c Kobe University, Japan

**Keywords:** wage growth, farm size, land rental, machine services, China

## Abstract

This paper aims to examine the dynamics of land transactions, machine investments, and the demand for machine services using farm panel data from China. Recently, China’s agriculture has experienced a large expansion of machine rentals and machine services provided by specialized agents, which has contributed to mechanization of agricultural production. On the other hand, the empirical results show that an increase in non-agricultural wage rates leads to expansion of self-cultivated land size. A rise in the proportion of non-agricultural income or the migration rate also increases the size of self-cultivated land. Interestingly, however, relatively educated farm households decrease the size of self-cultivated land, which suggests that relatively less educated farmers tend to specialize in farming. The demand for machine services has also increased if agricultural wage and migration rate increased over time, especially among relatively large farms. The results on crop income also support complementarities between rented-in land and machine services (demanded), which implies that scale economies are arising in Chinese agriculture with mechanization and active land rental markets.

## Highlights

• Rising real wages induced substitution of labor by machines in Chinese agriculture.
• An expansion of machine services by providers contributed to mechanization.
• Active land rental market enabled some farmers to increase their operational size.
• Relatively educated farmers tended to reduce their operational size.
• Scale economies are arising with mechanization and active land rental markets.

## 1 Introduction

China has made remarkable progress in increasing its income level through industrialization over the past three decades. It is also well known that, after the onset of reform, real wage rate for China’s unskilled labor had not risen for more than two decades, thereby supporting rapid industrialization (Cai & Du, 2011). However, during the past decade China’s unskilled wage rate appears to have been rising at an accelerating rate parallel to the GDP growth rate. It is common in the rural population that workers seek lucrative employment through migration to urban sectors, and the rising wage rate continues to encourage the rural–urban migration, which creates labor shortage in rural sectors. While the rising wage rate helps to reduce rural poverty, it is also creating an emerging challenge to agriculture in China, where production largely depends on small-scale, family-based and labor-intensive operations. In this paper, we examine (i) whether the use of machines (mainly through machine rental and services) substitutes for labor despite the prevalence of small-scale farming and land fragmentation and (ii) how the increase in real wages has induced a realization of scale economies through the use of agricultural machine and land rental markets under the institutional constraint of a prohibition on sale of agricultural land in China.

Family labor tends to be more intensively used on smaller farms in the absence of efficient labor markets due to difficulty in monitoring and supervising hire labor, which, in turn contributes to the inverse relationship between farm size and crop yield (Benjamin and Brandt, 2002, Berry and Cline, 1979, Chen et al., 2009, Feder, 1985). Chinese agriculture, dominated by labor-intensive small farms, mainly rely on family labor (Cook, 1999).^1^ However, such an inverse relationship could be changed when the economy grows fast, accompanying a rising real wage rate and thus making labor-intensive production expensive. The wage growth may have significant effects on the efficiency of small-scale farming in Asia and potentially more generally in land-scarce developing countries (Otsuka, Liu, & Yamauchi, 2013).

The following intuition shapes up the key hypotheses of this paper. An increase in real wages increases the production cost of labor-intensive farming system and thereby decreases comparative advantage in agriculture based on the labor-intensive production methods widely observed in many parts of Asia. To reduce the production cost, at least partially, farm size expansion helps mechanization to take place and therefore to substantially save high-cost labor, given that large machines are by nature indivisible. However, the introduction of mechanization would be difficult if farm size expansion is constrained by high transaction costs of land consolidation due to land fragmentation and/or imperfect land rental markets. China provides an interesting setting, in which selling agricultural land is prohibited and expansion of operational farm area can only be achieved through land rental markets. The prohibition on land sales creates an ideal experimental ground to assess how land rental markets respond to rising real wages.^2^

The key idea of this paper is related to the induced innovations proposed by Hicks (1932), and later elaborated by Hayami and Ruttan (1985) who introduced the idea of induced institutional changes in agriculture. An increase in real wages may induce a technical change to save labor or, simply, a substitution between labor and machines, i.e., mechanization, but also could lead to a new institutional arrangement that saves labor and/or reduces user costs of machines on farm even without land consolidation. As Otsuka (2013) elaborates, increasing real wages (and transformation of occupational structures in labor markets) challenge Asian agriculture in which the majority of farmers are smallholders, because of the increasing need (i) to reduce the labor force in agriculture (as the opportunity cost of labor increases), (ii) to increase the average farm size (to reduce labor use by introducing labor-saving production methods) and (iii) to generate enough income to retain parity with non-agricultural workers. If land markets and/or institutional mechanisms are imperfect, major inefficiencies in the allocation of farm land will be bound to arise. Otsuka *et al*. (2013) present evidence consistent with the above conjectures using cross-country panel data. Foster and Rosenzweig, 2010, Foster and Rosenzweig, 2011 show some evidence to support the second point in India. Yamauchi (2016) also shows evidence from Indonesia that relatively large farms gain more efficiency in production by expanding their farm land and introducing machines. Using commodity-wise province-level panel data, Wang, Yamauchi, and Huang (2014) showed clear evidence supporting the capital-labor substitution responding to changes in the relative price of machines to agricultural labor in China.

This paper shows evidence from China that largely supports the proposition that wage growth in recent years led to an introduction of labor saving practices. The emergence of machine service rental appears to mitigate the efficiency cost attributed to the land market rigidities in China by mitigating the indivisibility of machines. That is, prospective farmers tend to acquire more land by renting in land given the constraint of land sale market and also rely on machine services, rather than purchasing machines when real wages increase. This is especially true for relatively large farms. The empirical findings also show that land and machine services are complementary and its effect is larger and more significant among relatively large landholders.

The paper is organized as follows. Section 2 describes wage growth and mechanization recently observed in Chinese agriculture. Section 3 explains the panel data collected in six provinces in China, with two rounds in 2000 and 2008. Section 4 describes empirical strategy. The empirical findings are summarized in Section 5, followed by concluding remarks in Section 6.

## 2 Wage growth and mechanization in China

China’s economy has maintained its high annual growth rate of GDP, roughly at 10%, for more than four decades. In 2013, GDP per capita reached nearly US$6629 (NBSC, 2014). Among other factors, off-farm employment, especially through rural to urban labor migration, has played an important role in the nation’s structural transformation and has been a source of economic growth (Cai and Wang, 2010, Zhang and Song, 2013). The rise of off farm employment for the farm population was one of the most salient features of China’s development during the 1980s and 1990s. According to the 2000 China National Rural Survey only 15% of the rural labor force had a job off farm in the early 1980s. By 2000 the share of the rural labor force that worked off the farm reached 45.3% (Wang, Huang, Zhang, & Rozelle, 2011). With a rural labor force exceeding 500 million, this means that in 2000 more than 218 million individuals were working fully or part time off the farm (Giles, 2006, NBSC, 2001, 2013, 2014). The upward trend in the share of the rural labor force with off farm employment continues to rise. From 45.3% in 2000, more than 60% of the rural labor force is working off the farm in 2011.

When reviewing the process of adopting labor-saving technology in agriculture, it is generally assumed that this can be achieved through substituting machine-based engineering technology for labor. This, in turn, helps to save more labor time for nonfarm activities, potentially increasing income from other sources.

**Figure 1:**
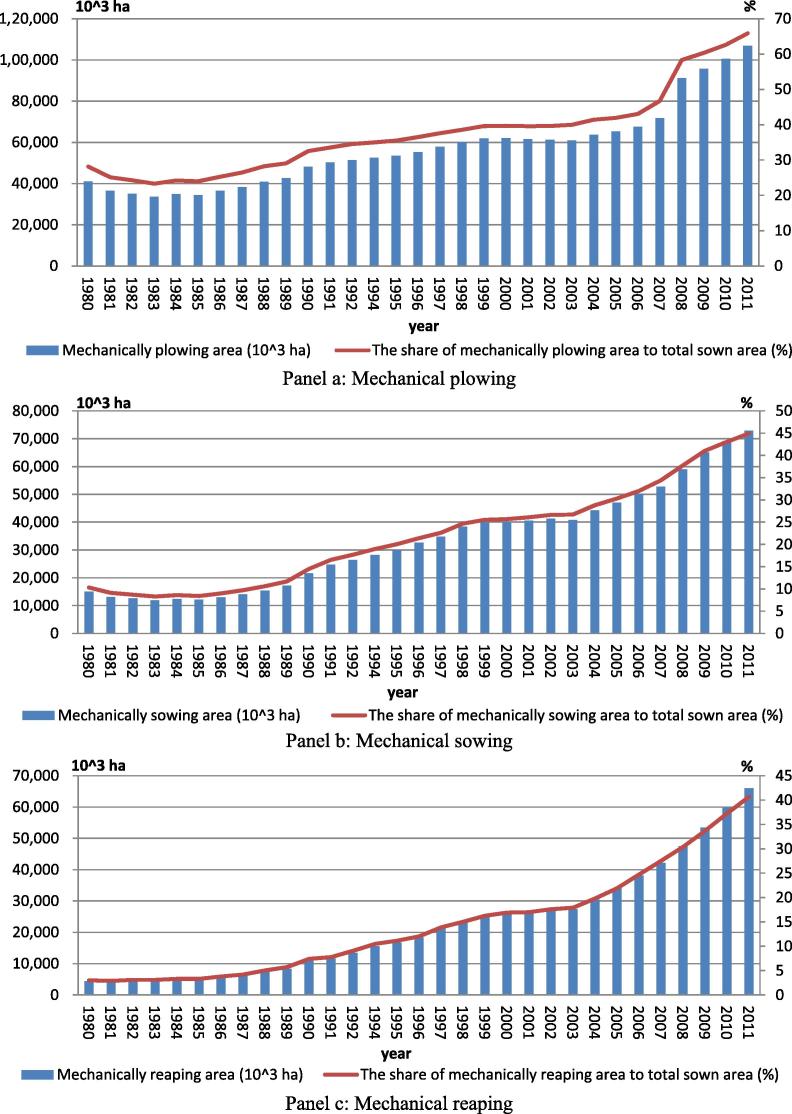
The evolution of mechanical farm operations in China’s agriculture.

*Source: China Rural Statistical Yearbook* (NBSC, 2012).

Initially, the effort to promote appropriate mechanization dates back to the stage of collective system before 1978. Even though large inefficiency is attributed to this collective institution, causing less motivated production and other adverse social effects, certain remarkable achievements have been acknowledged (Lin, 1991, Lin, 1992). Specifically, agricultural machinery stations at different administrative levels were established to provide machine operation services at the fixed price. Projects were designed to provide machine operations including plowing, sowing and reaping within villages, or production teams who were equipped with large- or medium-sized machines, especially tractors. This institutional mechanism also facilitated mutual aid among neighboring farmers to operate small motorized farming machines in peak seasons. As a result, mechanical farm operations increased gradually; about 28% of sown area were mechanically plowed in 1980 (Figure 1, Panel a).

The pattern of mechanical farm operations in Chinese agricultural production changed completely during the early period of rural reform. During the implementation of Household Responsibility System, small-sized machines and draft animals were distributed to households on an egalitarian basis. However, large- and medium-sized machines such as riding tractors which used to be shared by a production team composed of generally 20–30 households or managed by the committee of village leaders were not amenable to distribution to individual households. The use of mechanical operations in plowing declined because households sought to save operational costs and preferred to use draft animals for timely cultivations (Figure 1). From 1980 to 1983, more than 10 million ha of sown area were not mechanically plowed any more as it turned out not to be cost effective. The share of areas under which sowing and reaping were mechanically operated largely remained constant at 10% and 4%, respectively. Furthermore, the small size of cultivated land divided into several fragmented plots is another constraint that inhibits mechanical farm operations (Fleisher & Liu, 1992).

**Figure 2:**
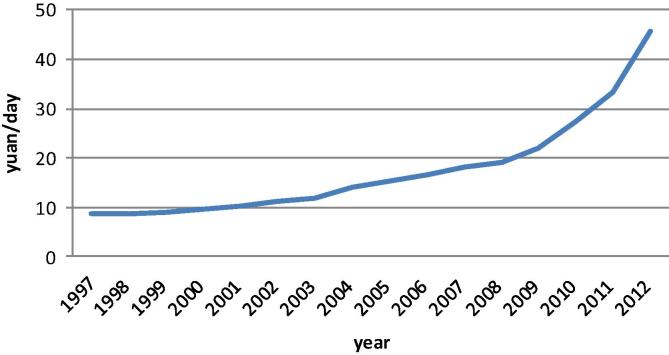
Trend of average daily wage rate of on-farm labor (yuan/day) in agricultural production, 1997–2012. *Note:* Cost is calculated at 2005 constant price.

*Source: National Compilation of Cost and Revenue in Agricultural Production* (National Development and Reform Commission, China, 1998–2013).

Experience in many developed countries shows that the process of mechanization is driven by changes in relative prices, particularly the rising wage rate of off-farm labor, and China is not an exception (Wang *et al*., 2014). The empirical studies by Cai, Park, and Zhao (2008), Wang *et al*. (2011) and Li, Li, Wu, and Xiong (2012) confirmed that migrant wages increased rapidly, along with wages available to other types of workers, since the late 1990s. The cost analysis in agricultural production also indicates that the annual growth rate of average on-farm labor cost (yuan/day, real term) was 8% during 1997–2008, and accelerated to reach more than 10% since (Figure 2). Under the pressure of rising on-farm labor costs and its opportunity cost determined by off farm employment, the number of days that China’s farmers have devoted to on-farm work has fallen significantly. By the mid-2000s, the average number of labor days per hectare spent on farm had fallen to less than half of the level in the 1990s (that is, less than 100 days per hectare) (de Brauw, Huang, Zhang, & Rozelle, 2013). In the past decade, the number of rural residents who have found off-farm employment has risen dramatically, and there has also been a sharp rise in the level of mechanization. The demand for mechanical farm operations has risen to compensate for the shortage of labor, especially in peak seasons, as the number of permanent migrants to urban and sub-urban areas has increased.

We can explore the adoption of mechanical farm operations from two dimensions: (i) the investment in agricultural machines by smallholders and (ii) the provision of machine services. Some earlier studies report positive correlations between investments in agricultural machines and remittances from migrants (de Brauw and Rozelle, 2008, Ji et al., 2012, Taylor et al., 2003). This suggests that, with the expansion of off farm employment opportunities that increase their incomes, smallholders whose household members work outside the village are more likely to substitute own agricultural machines for labor. Given the average farm size of 0.6 hectare with fragmented plots and possibly inflexible land rent markets, smallholders prefer to use small machines, such as less-than-12 horsepower tractors. In contrast to the US where the average farm size is around 180 hectares, Chinese smallholders cannot afford to use large machines to plow, plant, and harvest. It should be also noted that the frequency and scope of land reallocations negatively affects investments aimed to improve land productivity, because smallholders are afraid of losing such investments in case that they are assigned to different plots of land in the future without proper compensation for the initial investment (Zhang, Wang, Glauben, & Brümmer, 2011).^3^

The proportion of farmers investing in machines is low, which is partially explained by the availability of machine services across China, which in effect makes mechanical farm operations available to farmers without the indivisibility of exclusive reliance on owned machines. Even though land fragmentation still inhibits adoption of machines, Chinese smallholders have rapidly adopted machine rental services in many plain areas of China. The mechanization service by and large evolved spontaneously in response to emerging needs to substitute labor by capital (Liu & Wang, 2005).

Generally, two forms of mechanization are witnessed in the field. One is mechanical services provided by Specialized Custom Plowers, Planters and Harvesters (SCPPH) teams, who own large machines (Yang, Huang, Zhang, & Reardon, 2013). The other is machine rental markets, from which households can rent machines to operate on their farms. There has been a rapid rise in SCPPH teams’ activities. These teams are all private. They own machines and many of them are specialized in this activity; some do not even have their own contract land or have rented out their own contract land. Most typically, SCPPH teams are made up of two to three family members. Because agricultural production is still managed by smallholders, these teams generally set up an agreement orally or in writing on conditions such as price and time with all the households who cultivate one or several plots of land. They will provide mechanical operation services from plowing to harvesting to smallholders. The smallholders will come to the field to supervise the process of mechanical operations and to pay the machine service provider.^4^ Usually there is a “well-established price” for the services (that is, a kind of market price).

Mechanical operation teams have extended their activities beyond simply providing mechanical operation services. For example, in northeast China, these teams have started to rent in and consolidate land from smallholders to realize scale economies. Then they organize agricultural production with mechanical operations within the team and hire laborers as well. These teams typically also provide mechanical operation services to their neighboring farmers. They can use large-size machines on the consolidated land and upgrade their machines with subsidies provided from the government. However, they also face some constraints on keeping or expanding the consolidated land. First, the land rent-in contracts are mainly short term, often subject to renewal every year. The farmers who rent out their land expect that the rent will increase and thus hesitate to sign long-term contracts. The insecurity involved with using consolidated land (and, more generally, any short-term rental arrangements) make the operators less likely to invest in the land. Secondly, even though they would like to upgrade their machines, they may not able to obtain the quota to buy the subsidized large-size machines.^5^

Because of a move to off-farm employment, especially through migration, Chinese smallholders began to adopt mechanical farm operations to substitute for labor in production since the 1990s (Figure 1). The rapid expansion of mechanical operations occurred in plowing, sowing, and harvesting. Mechanically plowed areas doubled with an annual growth rate of more than 3% during 1983–2006 (Figure 1, Panel a).^6^ The growth of mechanically plowed areas accelerated to over 5% per year from 2008 to 2011. More than 72% of cultivated areas are now mechanically plowed. Mechanical sowing areas also doubled during the 1990s, but mechanically harvested areas increased only around 1.5 times (Figure 1, Panels b and c). Furthermore, mechanical sowing and reaping have started to accelerate since 2003 with annual growth rates of 4.5% and 7%, respectively. By 2011, more than 40% of cultivated areas are mechanically sown or reaped.

## 3 Data

### (a) Household survey

We use farm survey data that was collected in two rounds to represent the whole country. The Center for Chinese Agricultural Policy carried out the surveys in December 2000 (collecting data for the year 2000) and early 2009 (collecting data for the year 2008). The dataset for 2000 includes information from 60 randomly selected villages in six provinces representing China’s major agricultural regions. The selected provinces are Hebei, Liaoning, Shaanxi, Zhejiang, Sichuan and Hubei. A total of 1,200 households were sampled in the following way. For each province, five counties were selected. Then two villages were randomly selected from each county. Twenty farm households were chosen from each village. We judged that the data of 1,189 households out of the 1,200 initial sample households were complete. In the 2009 survey, we went back to the same villages that were surveyed in 2000. There were two exceptions. Because of the 2008 earthquake in Sichuan, we were not able to repeat the survey in two of the villages. As a consequence, the sample size (including those without complete records in 2001) was reduced from 1,200 to 1,160. Among the remaining 1,160 households surveyed in 2000, we were able to re-investigate 1,046 households in 2009. Of the 114 households that we could not find in the village, 89 had moved out of the village and were reported to be living in an urban area. The other 25 households either disappeared or were living in the village but were not engaged in farming activities (18 households—mostly because they were too sick to farm).

With special attention to crop production for this study, we constructed a panel dataset of households who were engaged in crop production. In the year 2000, among 1,194 sample households, around 90% of households (1,071 households) were engaged in crop production. Some households exited from crop production to allow more off-farm employment (Kimhi, 2000, Weiss, 1997). In the end, we use the panel dataset on crop production consisting of 905 households in the study.^7^

### (b) Agricultural production, landholding and machines

In this study, each household’s land endowment is captured by farm size and the number of plots. Farm size is measured as self-cultivated land, which is further decomposed into own land and net rent-in land. The net rent-in land is measured as land rented in either from the village or land rented in from other farmers minus land rented out.

In this study, the average self-cultivated land was 6.38 mu (0.42 ha) and 6.42 mu (0.43 ha) in 2000 and 2008 respectively, which was about 60% of farm size observed in 1985 (Table 1). Interestingly, the average size has been nearly constant from 2000 to 2008, the standard deviation has increased, which suggests that some farmers expanded their farm size. Our descriptive analysis shows that farm size was heterogeneous across provinces. In Hebei and Liaoning provinces, it was a bit larger than 1.5 times of the average size. On average, the number of plots was 4.61 and 4.09 in 2000 and 2008, respectively. Our data also showed a reduction of the number of plots in the sample provinces, except Hubei.

**Figure 3:**
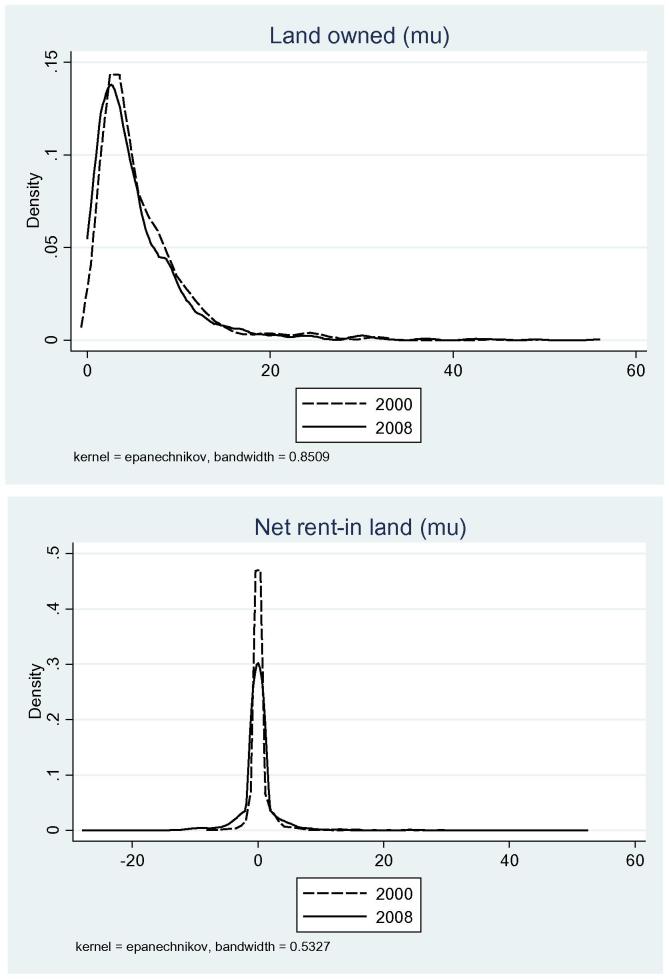
Kernel density estimation of land owned and net rent-in land (mu), 2000 and 2008.

**Figure 4:**
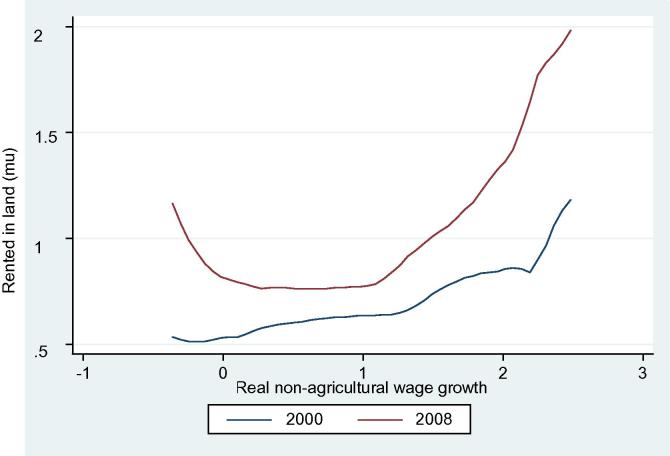
Rented in land and real non-agricultural wage growth.

The kernel density estimation of own land indicates that own land area did not change much during 2000–08 (Figure 3). This is consistent with the expectation that China has codified a robust framework for protection of land rights. Enlarging farm size could be achieved through more active utilization of land rental markets (Gao, Huang, & Rozelle, 2012). The distributions of net rent-in land show that average rent-in land increased during 2000–08. Figure 4 shows the relationship between rented in land and real non-agricultural wage growth (described below) in 2000 and 2008. The average size of land rented in has increased from 2000 to 2008. Besides, a positive relationship between rented in land and real non-agricultural wage growth is more visible in 2008 than 2000, which is consistent with our proposition.

Interestingly, we also observe that the area of land rented in has increased in the areas where non-agricultural wages are stagnant, which creates an u-shape curve. Migration from stagnant areas could be large as they head to high-growth areas in distance too, which may induce some farmers to rent in (as well as rent out) farmland.

The average number of plots ranged between 4 and 5.5 across provinces in 2000. Combined with our observations on farm size, this suggests that China’s agricultural production was facing the double pressures of small farm size and fragmentations. In the analysis, we include the number of plots in 2000 (and its squared term) as control variables.

**Figure 5:**
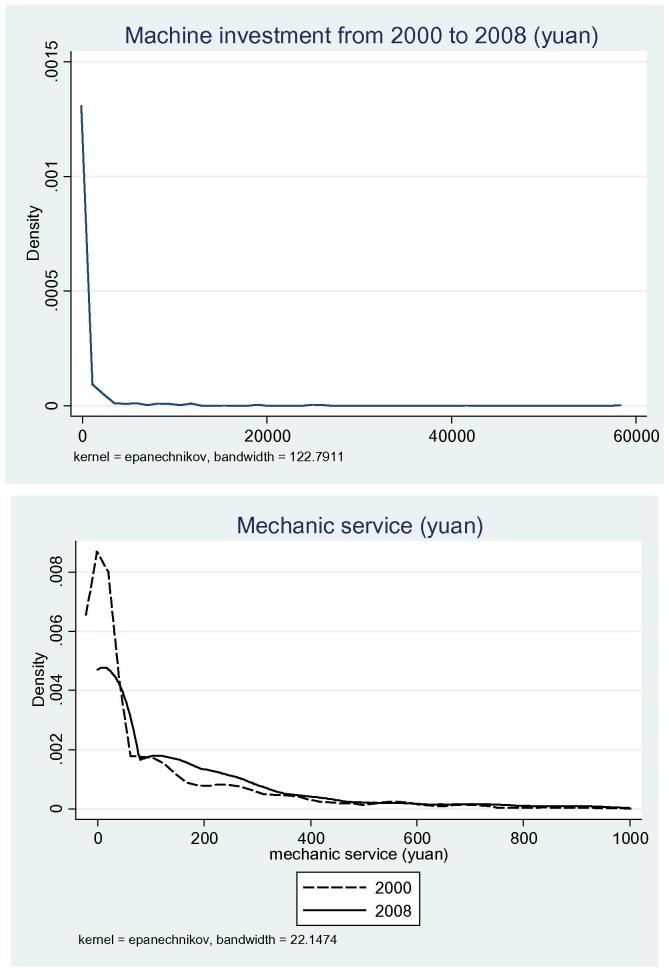
Kernel density estimation of investment in machinery and mechanical service (yuan), 2000 and 2008.

The quantities of both machine investment and demand for machine services are measured as purchases of machines and payments for machine services both in yuan at 2000 constant prices, respectively (Figure 5). Given their small farm size, it is not a surprise that smallholders are less likely to invest in machines. Among 905 rural households in the sample, about half did not invest in machines. About 15% invested less than 200 yuan (24 US$) in machines. These investments are typically in small tools such as pesticide sprayers, etc. The censored distribution of machine investments motivates us to use the Tobit model in Section 4.

The increased use of machine services could be found from the increased percentage of rural households who spent more on machine services (Figure 5). The percentage of rural households who rely on mechanical farm operation services increased from 49% in 2000 to 58% in 2008, with the average growth rate of 2%. Without adjusting for the price of machine services (yuan/mu), we found that the average expense increased from 217 yuan (26 US$) to 285 yuan (34 US$). This result is consistent with the national-level statistics that show the expansion of mechanical farm operations in plowing, planting, and harvesting.

### (c) Labor markets and wages

In this study, the key variables of labor supply and wage rates (agriculture and non-agriculture) are calculated at village level in order to mitigate the household-level endogeneity that jointly affects labor supply, wages, and productivity. Labor supply to off-farm employment is proxied by the proportion of off-farm income in total income for the sampled households in a village. On average, the proportion of off-farm income increased by 20.4 percentage point from 55.7% in 2000 to 75.1% in 2008. Furthermore, labor shortage in agriculture, especially those in peak seasons, could be captured by the migration rate in a village, which is defined as the proportion of household members who lived away (migrants) out of total laborers for all of the sampled households in a village. The migration rate doubled from 14.22% in 2000 to 28.62% in 2008, reflecting the rapid urbanization in China.

Wage rates used in this study are the average agricultural and non-agricultural wages in a village. The former is calculated from the cost of hired-in on-farm labor and the number of working days (yuan/day) for all of hired on-farm laborers in a village.^8^ The latter is the average wage for all off-farm workers in a village (yuan/hour), which is expected to reflect the opportunity cost of farm work in the local economy. Note that workers can find off-farm jobs not only in their village but also in the local towns and cities outside the village. Here, all of the value terms are adjusted at 2000 constant price using provincial CPIs.

Our analysis shows that the average real agricultural wage increased from 26.54 yuan/day (3.20 US$/day) in 2000 to 35.09 yuan/day (4.22 US$/day) in 2008 with an average annual growth rate of 3.5%. The kernel density of agricultural wage rates indicates that the average agricultural wage increased as its distribution moved from left to right during the period but quite mildly (Figure6a). Non-agricultural wages increased significantly from 2000 to 2008 at different growth rates (Figure6b). Our data show that hourly non-agricultural wages doubled from 1.92 yuan/h (0.23 US$/h) in 2000 to 4.00 yuan/h (0.48 US$/h) in 2008. This also suggests that, similar to those of agricultural wages, non-agricultural wages also present regional variations in 2000 and 2008.

**Figure 7:**
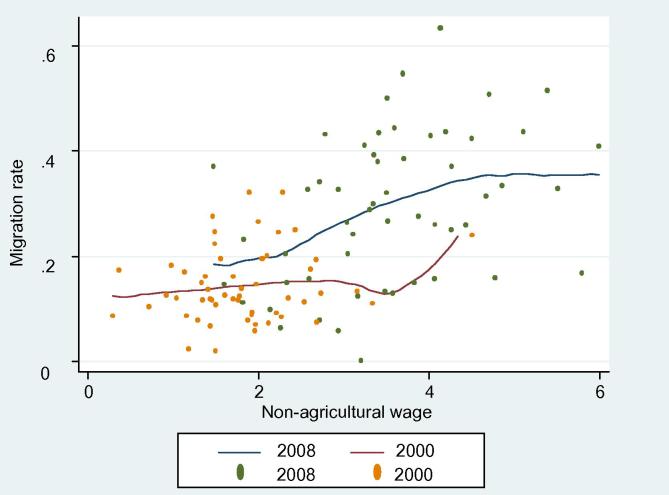
Migration rate and real non-agricultural wages (village-level).

Figure 7 plots village-level averages of migration rate and non-agricultural real wage rates in 2000 and 2008 (using provincial CPIs as deflators). Consistent with the Figure6b, it is clear that non-agricultural wages increased from 2000 to 2008. Migration rates also increased accordingly and are more responsible to non-agricultural wages in 2008 than 2000. Labor outmigration seems to be positively correlated with an increase in non-agricultural wages.

## 4 Empirical strategy

This section describes the specification and estimation strategy used, and discusses identification issues. The analysis uses household-level panel data to examine land transactions, machine services, and crop incomes. In the analysis of land transactions and demand for machine services, we investigate the effects of wage growth, both agricultural and non-agricultural, which potentially depends on the initial conditions such as landholding and human capital. Human capital, here represented by the average years of schooling completed in the household, determine non-agricultural labor market opportunities when wages increase. In contrast, the availability of relatively large farm land determines their comparative advantage in agriculture.

In all the econometric estimations, first differences are taken to wipe out unobserved fixed error components, which could lead to bias in the cross-sectional estimation. The key explanatory variable in the first-differenced form is village-level real wage growth separately computed for agriculture and non-agriculture work (as described in Section 3(c)). Labor can be imperfectly substitutable between agricultural and non-agricultural work due to differences in the required skills, and so we use the village-level wages for the two sectors. Furthermore, we use the proportion of non-agricultural income and migration rate, both computed at the village level, to represent labor shortage in agriculture. Finally, the analysis of crop income aims to investigate potential complementarities between machine services and land—either own or rent-in.

In the analysis of land transactions and machine investment and services, the following first-differenced equation is estimated,

(1) $$\Delta y_{\mathrm{ij}(0\text{,}1)}=\alpha +\beta_{1}\Delta w_{j(0\text{,}1)}+\beta_{21}\Delta w_{j(0\text{,}1)}{\mathrm{land}}_{\mathrm{ij}0}+\beta_{22}\Delta w_{j(0\text{,}1)}{\mathrm{edu}}_{\mathrm{ij}0}+x_{\mathrm{ij}0}^{\prime }\delta +{\mathrm{prov}}_{\mathrm{ij}}+\Delta \varepsilon_{\mathrm{ij}(0\text{,}1)}$$

where $\Delta y_{\mathrm{ij}(0\text{,}1)}$ is change in self-cultivated land or net rent-in land,^9^ or change in machine services purchased for household *i* in village *j*, during the period during 2000–08, $\Delta w_{j(0\text{,}1)}$ is the village-level real wage growth rate (agricultural and non-agricultural wages, treated separately), ${\mathrm{land}}_{\mathrm{ij}0}$ is the own-land or self-cultivated land size in 2000, ${\mathrm{edu}}_{\mathrm{ij}0}$ is the average years of schooling in 2000, $x_{\mathrm{ij}0}$ is a vector of initial household characteristics, ${\mathrm{prov}}_{\mathrm{ij}}$ is a province dummy, and $\Delta \varepsilon_{\mathrm{ij}(0\text{,}1)}$ is the difference in shocks (assume that $\varepsilon_{\mathrm{ijt}}$ is an ex-post shock after household decisions are made). Note that $\beta_{1}$ shows the effect of change in the village-level real wage rate on the dependent variable, and $\beta_{2}$ captures how the initial household characteristics affect the impact of change in the village-level real wage rate. In Eqn. (1) village-level real wage growth rate is interacted with the initial own or self-cultivated land size and the average years of schooling. In the estimation, we also include as $\Delta w_{j(0\text{,}1)}$ changes in the proportion of non-agricultural income and migration rate, both calculated at the village level.

We hypothesize that $\beta_{21}>0$ and $\beta_{22}<0\text{.}$. That is, facing rising real wages, relatively large holders tend to increase their operational size and invest in machines or increase their demand for machine services. On the other hand, relatively educated farmers who have better employment opportunities outside agriculture tend to reduce their operational size and, therefore, are reluctant to use machines.

We also estimate the crop income equation in the first differenced form:

(2) $$\Delta \ln\pi_{\mathrm{ij}(0\text{,}1)}=\alpha^{\prime }+\gamma_{1}\Delta {\mathrm{land}}_{j(0\text{,}1)}+\gamma_{2}\Delta {\mathrm{mach}}_{\mathrm{ij}0}+\gamma_{3}\Delta {\mathrm{land}}_{j(0\text{,}1)}\Delta {\mathrm{mach}}_{j(0\text{,}1)}+{\mathrm{village}}_{\mathrm{ij}}+\Delta \eta_{\mathrm{ij}(0\text{,}1)}$$

where $\Delta \ln\pi_{\mathrm{ij}(0\text{,}1)}$ is the crop income growth (that is, the difference in log of crop income, $\Delta {\mathrm{land}}_{j(0\text{,}1)}$ is change in the self-cultivated land, $\Delta {\mathrm{mach}}_{\mathrm{ij}0}$ is change in machine services purchased, ${\mathrm{village}}_{\mathrm{ij}}$ is village fixed effects, and $\Delta \eta_{\mathrm{ij}(0\text{,}1)}$ is the difference in ex-post shocks. The variable $\Delta {\mathrm{land}}_{j(0\text{,}1)}$ can be decomposed into changes in own land and net rent-in land. Note that log transformation, once combined with location (village) dummies, purges common-unit effects such as price and location-specific shocks. Therefore, village-level common shocks and price changes (specific to village) are controlled. The estimation uses instruments to remove potential bias due to the correlations between initial period shock and changes in land and machine inputs.^10^ Since village fixed effects are included, the inference is based on intra-village variations. Drawing upon the results in Eqn. (1), the instruments are the interaction terms of (i) village-level non-agricultural real wage growth, agricultural real wage growth, change in non-agricultural incomes, and migration rate, (ii) the initial own land size and the average years of schooling, and (iii) province dummies. The interaction of (i) and (ii) creates household-level variations. Heterogeneity in their effects is introduced across provinces by interacting them with (iii) province dummies. Since we control village fixed effects, the village level variables themselves are not included in the instruments.

Our interest is in $\gamma_{3}$ measuring the complementarity between land and machines. In the context of China where the transfer of land ownership is prohibited, we are particularly interested in the role of land rental arrangements in expanding (or reducing) the size of self-cultivated land and realizing scale economies by augmenting the marginal value of machines. That is, we hypothesize that $\gamma_{3}>0$.

## 5 Empirical results

This section reports our empirical results. The first set of estimation results focuses on land transactions, machine investments, and machine services demanded. We use first differencing in all estimations. The next set comes from crop income equations. Instruments are used to endogenize changes in land cultivated and rented in as well as machine investments and services demanded in first differenced form.

Table 2 summarizes our results on changes in self-cultivated farm land.^11^ The explanatory variables include non-agricultural real wage growth, agricultural real wage growth, change in the proportion of non-agricultural income, and the migration rate, all of which were computed at the village level. The village-level changes are interacted with the household’s land owned in 2000 and the average years of schooling completed (as of 2000). These interaction terms are intended to capture the degree to which the initial levels of household land and human capital endowment differentiate the effects of the village-level changes. In addition, the specifications include, as controls, the number of plots, its squared term, land owned, the number of laborers, that of family laborers, the average age, and years of schooling completed. Province dummies are also included to control for the province-level average changes.

Columns 1 and 2 include non-agricultural and agricultural real wage growth, both of which are interacted with the initial size of land owned and the average years of schooling completed. Column 1 has the number of plots, while Column 2 adds its squared term. The results show that non-agricultural real wage growth has a significant and positive effect on change in self-cultivated land, implying that cultivated land area has increased significantly in villages that experienced an increase in real wage in non-agricultural sectors. In contrast, we do not find significant effects of agricultural real wage growth.

Columns 3 and 4 also add changes in non-agricultural income and migration rate (both computed at the village level), interacted with the initial size of land owned and the average years of schooling completed. First, the effects of non-agricultural real wage growth remain robust. Second, change in the proportion of non-agricultural income also has a significant and positive effect on change in self-cultivated land. Third, their interactions with the average years of schooling has significant and negative effects, which implies that, in response to employment opportunities in non-agricultural sectors, farm households endowed with more human capital (measured in educational attainment) tend to reduce the size of farm operations by renting out land. Finally, the negative sign of change in migration rate is hard to interpret here but this could be because large migration out of the village could shrink agricultural activities.^12^

Table 3 reports the estimation results on change in net rent-in land. The net rent-in land is land rented in minus that rented out. Consistent with the previous findings, non-agricultural real wage growth has a significant and positive effect on change in net rent-in land. Its interaction term with the average years of schooling is also negative and significant. Farmers tend to rent in land when the non-agricultural wage increases, but rent out land if the households have more educated members. Other variables of interest are not statistically significant.

Next we analyze machine investments and service demands. Table 4 shows the results on machine investments. The aggregate value of investments in agricultural machines in 2000–08 was computed. The estimation uses the same specifications as in Table 2, Table 3, but we use the Tobit model for machine investments because the dependent variable is censored at zero (nearly a half of observations have no investment). Only the interaction of agricultural real wage growth with the initial size of self-cultivated land (Columns 1 and 2) and the initial size of land owned (all columns) are significant. We may conclude that machine investments are explained primarily by the initial cultivation size and growth of agricultural wages, but not by changes in non-agricultural employment opportunities in the current empirical context.

Table 5 shows the results on machine services demanded. First, similar to the results on machine investments, an increase in agricultural real wage raises the value of machine services demanded if the size of self-cultivated land is relatively large. Second, an increase in migration rate also raises the demand for machine services when self-cultivated land is relatively large at the initial stage. Both results imply that the demand for machine services increases as it becomes more difficult and/or expensive to secure labor for agricultural operations.

Next we check robustness of our results using village fixed effects. Table 6 shows village fixed effects estimation results on changes in self-cultivated land, net rent-in land and machine services. The results on machine investments were omitted as the results were insignificant as previously shown in Table 4. The specifications do not include linear terms of wage growth (both agricultural and non-agricultural) and changes in the proportion of non-agricultural income and migration rate, all calculated at the village level, since their effects were controlled by village fixed effects. These are consistent with our previous findings that in the self-cultivated land equations (Columns 1 and 2), the education effects were significantly negative with non-agricultural wage growth and change of the non-agricultural income proportion. Similarly, the initial land effect is significantly positive with agricultural wage growth in the net rent-in equation (Column 3) and the machine service equation (Column 5). Though significance level varies across equations, the heterogeneous effects by the initial landholding and educational attainment remain robust, consistent with the results obtained with province dummies.

Table 9 checks the education effect using a sub-sample of households that had the number of out-migrants in 2008 smaller than or equal to that of 2000. Note that those households might have changed out-migrants but the number of out-migrants stayed the same or became smaller in 2008, which indicates that their net migration after 2000 was zero or negative. Interestingly, the results remain qualitatively the same in the self-cultivated land and net rent-in land equations, implying that even if relatively educated farmers stay farming, they tend to reduce the operational size in response to rising real wages.

Overall, the results on land transactions and demands for machines are consistent. Non-agricultural wage growth and larger employment opportunities in non-agricultural sectors tend to lead to increases in the size of farm operations by renting in more land, and this effect seems to be larger among relatively large farms. In contrast, the above effect is negative if more educated members are in the household, most likely because those households want to allocate relatively educated members to non-agricultural jobs. Machine investments and services demanded also tend to increase in response to an increase in agricultural wage (not non-agricultural wage), and the effect seems to be large among relatively large farms. Out migration also tends to increase the demand for machine services among relatively large farms, which directly supports the substitution for labor by machines.

Now we report results on crop income equations. The dependent variable is growth of crop income (crop revenues minus all the production costs except family labor and other family-owned inputs) in the first differenced forms with village fixed effects (thus, making it unit free). We estimate the equations with and without instruments. Based on our previous results, the identifying instruments are the interaction terms of (i) village-level non-agricultural real wage growth, agricultural real wage growth, change in non-agricultural incomes, and migration rate, (ii) the initial own land or the average years of schooling and (iii) province dummies. The interaction of (i) and (ii) creates household-level variations and their marginal effects vary by province. Since we control village fixed effects at both the first and second stages, the village-level variables themselves are not included in the instruments. Essentially, the first stage analysis utilizes the results from Table 2, Table 5.

The results in Table 7 confirm significant effects of land cultivated on income but not the effect of machine services. These results remain robust whether they are estimated with instruments or not. The estimation also separates the cultivated land into the land owned and rented in (measured by change in net rent-in land). As expected, both types of land significantly contribute to crop income. The interaction of self–cultivated land (or net rent-in land) and machine services is significantly negative without instruments. The interaction of changes in net rent-in land and machine services is significantly positive in the instrumental variable estimation. Machine services seem to be complementary with rent-in land. In the above estimations, however, Hausman tests did not detect significant differences in estimates between non-instrument and instrumental variable estimations.

In Columns 5–8, we restrict the sample to farmers who own land greater than 6 mu in 2000 (relatively large farmers). In all specifications, Hausman tests support the instrumental variable estimation results, which justify us to focus on the results with instruments. Although machine services are not significant without interactions with land, they are significantly complementary with rent-in land. The parameter is larger than the previous estimate.^13^

To sum up, the crop income equations show that (i) land, owned and rented in, significantly contributes to crop income and (ii) the contribution of machines to crop income depends on land types and sizes, and in particular, machine services seem to augment the value of rent in land, but not own land, indicating that land is rented into enhance the efficiency of machine use. The results are more clearly interpretable among relatively large farms. That is, rent in land and machine services are complementary for large farmers. This finding implies that the possibility of renting in land to expand the scale of farm operation as well as the availability of machine services that substitute for labor are particularly important among relatively large farms. In other words, the advantage of large-scale farming is realized by the increasing incidence of land renting and the increasing availability of machine services.

## 6 Conclusions

Using farm panel data from China, collected in six provinces, i.e., Hebei, Hubei, Liaoning, Shaanxi, Sichuan and Zhejiang, in 2000 and 2008, we examined dynamics of land transactions, machine investments and the demand for machine services. China’s agriculture in general experienced an expansion of machine rentals and machine services provided by specialized agents in the past decade, which contributed to mechanization in agricultural production. In particular, we investigated the effects of non-agricultural and agricultural wage growth and changes in the proportion of non-agricultural income and migration rate, all of which are estimated at the village level, on changes in self-cultivated land, rent-in land, machine investments and machine service used. Our results show that an increase in non-agricultural wage leads to the expansion of self-cultivated land size, and the effect tends to be larger among larger farms. A rise in the proportion of non-agricultural income or migration rate also significantly increases the size of self-cultivated land among relatively large farms.

Interestingly, our results also show that relatively educated farm households respond to the above changes in an opposite way, i.e., decreasing the size of self-cultivated land, which suggests that land is rented out from relatively more educated to less educated households. Since the initial land distribution is relatively equal for historical reasons, it is schooling distribution across households that seem to play a more important role in differentiating households: those who rent in land to expand farm size and those who transit to non-agricultural works and rent out land to others. This finding is in contrast to those found in Indonesia where the initial landholding plays an important role (Yamauchi, 2016).

The demand for machine services has also increased if agricultural wage and migration rate increased over time, and the effect is larger among relatively large farms. In contrast, machine investments were not responding to wage growth possibly because of the development of active machine rental and service markets (Yang *et al*., 2013). Interestingly, the results on crop income equations support the complementarity between rent-in land and machine services (demanded), both of which are mutually augmenting crop income. The possibility of renting in land to expand farm size and the availability of machine service providers or machine rental markets are both critically important to enhance the efficiency of large farms.

The above findings largely support our main hypothesis that wage growth, now increasingly important as a result of the successful industrialization in China, creates pressure on farmers to substitute labor by machine services as well as expand the scale of farm operations. In order to do so, the land institutions in China need to be flexible enough to allow the emergence of larger scale farms, which will help to maintain the international competitiveness of Chinese agriculture. Conversely, if their land institutions fail to support the emergence of larger scale farms, Chinese agriculture is likely to lose the comparative advantage.

## Figures and Tables

**Table 8:** Attrition analysis

Dependent variable: 1 = if attrition in 2008 and 0 otherwise Sample: Households in 2000	
Real wage growth: Non ag	−0.0384
	(0.15)
Real wage growth: Non ag * Land owned	0.0040
	(0.26)
Real wage growth: Non ag * Years of schooling	−0.0250
	(0.69)
Real wage growth: Ag	0.0695
	(0.33)
Real wage growth: Ag * Land owned	0.0051
	(0.40)
Real wage growth: Ag * Years of schooling	−0.0405
	(1.42)
Change in the proportion of non agri income	−0.3796
	(0.67)
Change in the proportion of non agri. income * Land owned	−0.0216
	(0.39)
Change in the proportion of non agri. income * Years of schooling	0.0638
	(0.80)
Change in migration rate	1.7122
	(1.41)
Change in migration rate * Land owned	−0.1869
	(1.40)
Change in migration rate * Years of schooling	0.0730
	(0.47)
No. of plots (2000)	−0.7440^∗∗∗^
	(12.22)
No. of plots^2^ (2000)	0.0604^∗∗∗^
	(10.23)
Land owned (2000)	0.0596^∗∗^
	(2.12)
Number of labors (2000)	−0.2231^∗∗∗^
	(3.80)
Female (labor, 2000)	0.0341
	(0.52)
Age (labor, 2000)	−0.0083
	(1.42)
Years of schooling (labor, 2000)	−0.0136
	(0.31)
Net crop income at 2000 (yuan)	0.0000
	(0.07)
*N*	1126
*R*^2^	0.2352

*Note:* Absolute *t* statistics in parentheses; ^*^p < 0.10, ^**^p < 0.05, ^***^p < 0.01.

**Table 9:** Households with the number of out-migrants in 2008 equal to or smaller than that of 2000

Dependent variable:	Self-cultivated land (mu)		Net rent-in land (mu)	
Real wage growth: Non ag	0.8541^∗^	0.9624^∗^	0.8189	0.8860
	(2.11)	(2.34)	(1.45)	(1.84)
Real wage growth: Non ag * Self-cultivated land	0.0479	0.1190	0.0098	0.0263
	(0.89)	(1.66)	(0.18)	(0.39)
Real wage growth: Non ag * Years of schooling	−0.1582	−0.2356^∗∗∗^	−0.1145	−0.1310^∗^
	(1.82)	(4.28)	(1.40)	(2.55)
Real wage growth: Ag	0.5822	0.8488	−0.7662	−0.8734
	(1.34)	(1.64)	(1.82)	(1.42)
Real wage growth: Ag * Self-cultivated land	−0.0162	−0.0006	0.0078	0.0202
	(0.37)	(0.01)	(0.18)	(0.29)
Real wage growth: Ag * Years of schooling	−0.1281	−0.1740	0.0641	0.0596
	(1.34)	(1.66)	(1.43)	(1.33)
Change in the proportion of non agri income		2.9824^∗∗^		−0.2217
		(2.62)		(0.26)
Change in the prop of non agri income * Self-cult land		0.2097		0.0739
		(1.00)		(0.47)
Change in the prop of non agri income^∗^Yrs of schooling		−0.5515^∗∗∗^		−0.1473
		(5.18)		(1.17)
Change in migration rate		−0.0288		−1.7763
		(0.01)		(0.76)
Change in migration rate * Self-cultivated land		0.5712		0.2484
		(0.84)		(0.32)
Change in migration rate * Years of schooling		0.2687		0.1395
		(0.83)		(0.37)
No. of plot	−0.5866^∗^	−0.5977^∗^	−0.7341^∗∗∗^	−0.7395^∗∗∗^
	(2.09)	(2.11)	(4.20)	(4.21)
No. of plot^2^	0.0653^∗^	0.0622^∗^	0.0654^∗∗^	0.0663^∗∗^
	(2.16)	(2.02)	(3.32)	(3.25)
Land owned (2000)	−0.2656^∗^	−0.4522^∗^	0.0019	−0.0599
	(2.13)	(2.49)	(0.02)	(0.38)
Number of labors (2000)	−0.3167	−0.3087	0.0561	0.0514
	(0.95)	(0.94)	(0.27)	(0.24)
Female (labor, 2000)	0.1397	0.1888	0.0312	0.0359
	(0.48)	(0.72)	(0.14)	(0.17)
Age (labor, 2000)	−0.0433^∗∗^	−0.0384^∗∗^	0.0012	0.0017
	(3.27)	(3.32)	(0.09)	(0.13)
Years of schooling (labor, 2000)	0.0782	0.2374	0.1256	0.1504^∗∗^
	(0.76)	(1.82)	(1.94)	(2.81)
Net crop income (yuan, 2000)	0.0000	−0.0000	0.0000	0.0000
	(0.05)	(1.26)	(0.32)	(0.39)
Constant	3.4603^∗∗^	3.4249^∗∗^	−0.8544	−0.5248
	(2.96)	(2.72)	(0.62)	(0.32)
Province dummies	Yes	Yes	Yes	Yes
*N*	720	720	720	720
*R*^2^	0.116	0.139	0.057	0.060

*Note:* Absolute *t* statistics in parentheses; ^*^p < 0.10, ^**^p < 0.05, ^***^p < 0.01.

**Figure 6a:**
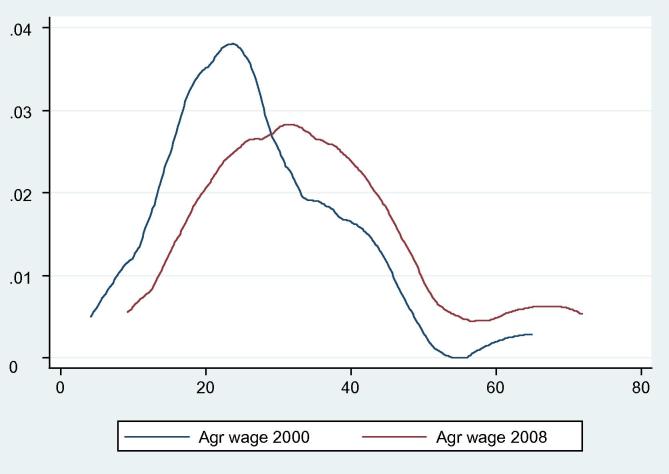
Kernel densities of real agricultural wage (yuan/day) in 2000 and 2008

**Figure 6b:**
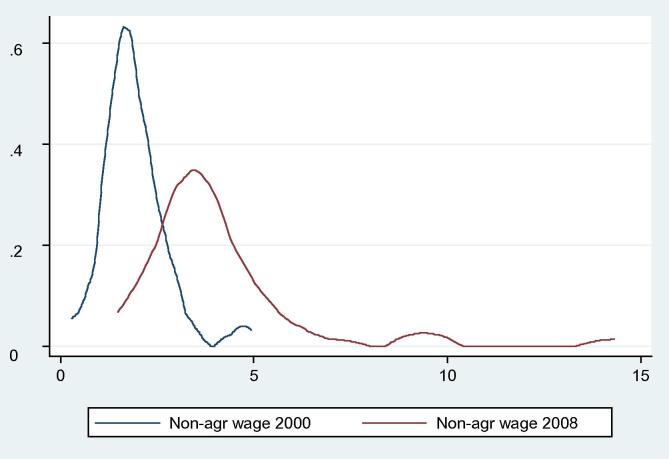
Kernel densities of real non-agricultural wage (yuan/hour) in 2000 and 2008.

**Table 1:** Descriptive statistics of self-cultivated land and no. of plots by province, 2000 and 2008

Provinces	Self-cultivated land (mu)		Number of plots (no.)	
2000	2008	2000	2008
All provinces	6.38	6.47	4.61	4.09
(6.01)	(15.41)	(2.16)	(2.23)
Hebei	10.82	10.20	4.47	3.33
(9.03)	(18.97)	(2.13)	(1.83)
Shaanxi	5.63	4.52	3.98	3.00
(2.94)	(3.21)	(1.87)	(1.74)
Liaoning	9.97	12.21	5.18	4.50
(7.06)	(28.27)	(2.40)	(2.23)
Zhejiang	3.39	3.68	4.23	3.81
(2.19)	(8.59)	(1.92)	(2.13)
Sichuan	3.65	2.40	5.44	5.32
(1.96)	(2.28)	(2.07)	(2.45)
Hubei	4.31	4.76	4.33	4.86
(3.27)	(5.93)	(2.13)	(2.13)

*Source:* Authors’ own survey.

**Table 2:** Determinants of change in self-cultivated land

Dependent variable: Change in self-cultivated land (mu)				
Real wage growth: Non ag	1.1010^∗^	1.0826^∗^	1.2566^∗^	1.2586^∗^
	(2.48)	(2.43)	(2.30)	(2.24)
Real wage growth: Non ag * Land owned (2000)	−0.0020	0.0062	0.0879	0.0908
	(0.04)	(0.12)	(1.23)	(1.29)
Real wage growth: Non ag * Years of schooling (labor, 2000)	−0.1625	−0.1712	−0.2661^∗^	−0.2730^∗^
	(1.31)	(1.36)	(2.24)	(2.29)
Real wage growth: Ag	0.7126	0.6743	1.2256	1.1759
	(1.25)	(1.17)	(1.54)	(1.41)
Real wage growth: Ag * Land owned (2000)	−0.0399	−0.0391	−0.0351	−0.0360
	(1.15)	(1.13)	(0.83)	(0.86)
Real wage growth: Ag * Years of schooling (labors, 2000)	−0.0970	−0.0882	−0.1573	−0.1487
	(1.04)	(0.92)	(1.33)	(1.22)
Change in the proportion of non agri income			3.3211^∗∗^	3.2183^∗∗^
			(3.68)	(3.78)
Change in the proportion of non agri. income * Land owned (2000)			0.3048	0.3055
			(1.40)	(1.38)
Change in the proportion of non agri. income * Years of schooling (labors, 2000)			−0.5190^∗∗^	−0.5230^∗∗^
			(3.59)	(3.78)
Change in migration rate			−2.2392^∗^	−1.8656^∗^
			(2.18)	(2.25)
Change in migration rate * Land owned (2000)			0.5273	0.4724
			(0.94)	(0.85)
Change in migration rate * Years of schooling (labors, 2000)			0.3693	0.3760
			(0.95)	(1.00)
No. of plots (2000)	−0.0248	−0.5575^∗^	−0.0624	−0.5489^∗∗^
	(0.16)	(2.53)	(0.42)	(2.60)
No. of plots^2^ (2000)		0.0518^∗∗^		0.0474^∗∗^
		(3.53)		(3.42)
Land owned (2000)	−0.1667	−0.1694	−0.3923^∗^	−0.3839^∗^
	(1.41)	(1.44)	(2.24)	(2.19)
Number of labors (2000)	−0.3893	−0.3702	−0.3551	−0.3373
	(1.67)	(1.64)	(1.54)	(1.50)
Female (labor, 2000)	0.0954	0.0855	0.1375	0.1299
	(0.34)	(0.31)	(0.51)	(0.48)
Age (labor, 2000)	−0.0649^∗∗^	−0.0653^∗∗^	−0.0585^∗∗^	−0.0591^∗∗^
	(2.83)	(2.93)	(2.77)	(2.88)
Years of schooling (labor, 2000)	0.0600	0.0549	0.2151	0.2095
	(0.70)	(0.62)	(1.72)	(1.69)
Net crop income (yuan, 2000)	0.0000	0.0000	−0.0000	−0.0000
	(0.14)	(0.20)	(1.25)	(1.16)
Province dummies	Yes	Yes	Yes	Yes
*N*	905	905	905	905
*R*^2^	0.081	0.084	0.100	0.103

*Note:* Absolute *t* statistics in parentheses; ^*^p < 0.10, ^**^p < 0.05, ^***^p < 0.01.

**Table 3:** Determinants of change in net rent-in land

Dependent variable: Change in net rent-in land (mu)				
Real wage growth: Non ag	0.7814^∗∗^	0.7656^∗∗^	0.7950^∗^	0.7969^∗^
	(2.61)	(2.85)	(2.08)	(2.11)
Real wage growth: Non ag * Land owned (2000)	0.0069	0.0139	0.0271	0.0298
	(0.25)	(0.50)	(0.51)	(0.57)
Real wage growth: Non ag * Years of schooling (labor, 2000)	−0.1097^∗^	−0.1172^∗^	−0.1259^∗∗∗^	−0.1322^∗∗∗∗^
	(2.20)	(2.44)	(4.13)	(4.46)
Real wage growth: Ag	−0.7135	−0.7463	−0.6941	−0.7396
	(1.81)	(1.95)	(1.06)	(1.18)
Real wage growth: Ag * Land owned (2000)	0.0123	0.0130	0.0177	0.0169
	(0.45)	(0.47)	(0.31)	(0.30)
Real wage growth: Ag * Years of schooling (labor, 2000)	0.0622	0.0698	0.0573	0.0652
	(1.36)	(1.60)	(0.97)	(1.17)
Change in the proportion of non agri income			0.1049	0.0107
			(0.07)	(0.01)
Change in the proportion of non agri. income * Land owned (2000)			0.0686	0.0692
			(0.53)	(0.55)
Change in the proportion of non agri. income * Years of schooling (labors, 2000)			−0.0740	−0.0777
			(0.36)	(0.39)
Change in migration rate			−1.8823	−1.5403
			(0.72)	(0.71)
Change in migration rate * Land owned (2000)			0.1955	0.1452
			(0.33)	(0.24)
Change in migration rate * Years of schooling (labors, 2000)			0.0599	0.0660
			(0.12)	(0.13)
No. of plot (2000)	−0.0590	−0.5149^∗∗∗^	−0.0530	−0.4984^∗∗∗^
	(0.70)	(6.72)	(0.59)	(6.97)
No. of plot^2^ (2000)		0.0444^∗∗∗∗^		0.0433^∗∗∗^
		(5.38)		(4.86)
Land owned (2000)	0.0095	0.0072	−0.0498	−0.0421
	(0.13)	(0.10)	(0.41)	(0.35)
Number of labors (2000)	−0.0110	0.0053	−0.0137	0.0026
	(0.07)	(0.03)	(0.08)	(0.02)
Female (labor, 2000)	0.1196	0.1111	0.1194	0.1124
	(0.55)	(0.52)	(0.54)	(0.51)
Age (labor, 2000)	−0.0063	−0.0067	−0.0056	−0.0062
	(0.58)	(0.61)	(0.51)	(0.55)
Years of schooling (labor, 2000)	0.1144^∗^	0.1101^∗^	0.1331^∗∗^	0.1280^∗∗^
	(2.49)	(2.37)	(2.88)	(2.96)
Net crop income (yuan, 2000)	0.0000	0.0000	0.0000	0.0000
	(0.57)	(0.62)	(0.54)	(0.61)
Province dummies	Yes	Yes	Yes	Yes
*N*	905	905	905	905
*R*^2^	0.034	0.039	0.035	0.040

*Note:* Absolute *t* statistics in parentheses; ^*^p < 0.10, ^**^p < 0.05, ^***^p < 0.01.

**Table 4:** Determinants of machine investments

Dependent variable: Machine investment (yuan)				
Real wage growth: Non ag	1,646.2786	1,604.7218	2,498.9604	2,462.9412
	(0.72)	(0.70)	(1.07)	(1.05)
Real wage growth: Non ag * Self-cultivated land (2000)	−38.6778	−25.6026	−52.0189	−42.0548
	(0.89)	(0.71)	(1.10)	(0.99)
Real wage growth: Non ag * Years of schooling (labor, 2000)	−50.3236	−63.5197	−164.6159	−177.0121
	(0.21)	(0.25)	(0.80)	(0.82)
Real wage growth: Ag	−322.4894	−400.0418	233.1681	135.0249
	(0.28)	(0.35)	(0.25)	(0.15)
Real wage growth: Ag * Self-cultivated land (2000)	96.9997^∗∗∗^	97.2777^∗∗∗^	33.8736	32.3809
	(2.63)	(2.71)	(0.75)	(0.68)
Real wage growth: Ag * Years of schooling (labor, 2000)	94.6228	114.6717	55.6599	76.0612
	(0.58)	(0.72)	(0.36)	(0.52)
Change in the proportion of non agri income			3,006.4947	2,808.0877
			(0.70)	(0.68)
Change in the proportion of non agri income * Self-cultivated land (2000)			380.4257	385.2402
			(1.42)	(1.47)
Change in the proportion of non agri income * Years of schooling (labor, 2000)			−774.7139	−774.1202
			(1.39)	(1.40)
Change in migration rate			−6,155.0801	−5,382.7883
			(0.50)	(0.45)
Change in migration rate * Self-cultivated land (2000)			−752.7662	−841.7403
			(1.39)	(1.59)
Change in migration rate * Years of schooling (labors, 2000)			1,038.3203	1,028.3972
			(0.87)	(0.87)
No. of plot (2000)	−227.4205	−1,164.7252	−167.9951	−1,131.2169^∗^
	(1.23)	(1.63)	(1.08)	(1.78)
No. of plot^2^ (2000)		89.5733		92.2336^∗^
		(1.61)		(1.77)
Land owned (2000)	137.4507^∗∗∗^	133.7114^∗∗^	160.692^∗∗∗^	168.4947^∗∗∗^
	(2.66)	(2.37)	(3.03)	(3.08)
Number of labors (2000)	48.2392	82.1584	76.5001	111.8911
	(0.34)	(0.60)	(0.51)	(0.80)
Female (labor, 2000)	480.7778	465.4561	520.3242	506.8017
	(1.22)	(1.20)	(1.33)	(1.32)
Age (labor, 2000)	−65.1338	−67.0338	−63.4615	−65.6581
	(1.56)	(1.62)	(1.51)	(1.57)
Years of schooling (labor, 2000)	41.2211	27.5204	142.4075	128.7741
	(0.22)	(0.14)	(0.56)	(0.52)
Net crop income (yuan, 2000)	0.0720^∗∗∗^	0.0741^∗∗^	0.0855^∗∗^	0.0892^∗∗^
	(2.05)	(2.07)	(2.10)	(2.13)
Province dummies	Yes	Yes	Yes	Yes
Sigma	6,913.90^∗∗∗^	6,887.91^∗∗∗^	6,895.43^∗∗∗^	6,868.54^∗∗∗^
	(4.49)	(4.54)	(4.55)	(4.59)
*N*	905	905	905	905
Log likelihood	−5,007.524	−5,005.2744	−5,004.2946	−5,001.9182

*Note:* Absolute *t* statistics in parentheses; ^*^p < 0.10, ^**^p < 0.05, ^***^p < 0.01.

**Table 5:** Determinants of change in machine services

Dependent variable: Change in machine service (yuan)				
Real wage growth: Non ag	17.6465	18.2858	26.9568	27.1143
	(0.36)	(0.38)	(0.48)	(0.49)
Real wage growth: Non ag * Self-cultivated land (2000)	−0.7045	−0.9709	−1.4316	−1.5845
	(0.25)	(0.33)	(0.55)	(0.59)
Real wage growth: Non ag * Years of schooling (labor, 2000)	−2.0099	−1.6874	−1.1506	−0.8777
	(0.29)	(0.25)	(0.17)	(0.13)
Real wage growth: Ag	35.8991	37.2477	−8.0805	−6.0585
	(0.80)	(0.84)	(0.17)	(0.13)
Real wage growth: Ag * Self-cultivated land (2000)	3.3223^∗^	3.3254^∗^	7.8305^∗∗^	7.8239^∗∗^
	(2.23)	(2.22)	(3.96)	(3.84)
Real wage growth: Ag ^∗^Years of schooling (labors, 2000)	−6.4560	−6.8117	−4.7465	−5.0752
	(1.22)	(1.31)	(0.97)	(1.04)
Change in the proportion of non agri income			20.6135	23.2196
			(0.30)	(0.33)
Change in the proportion of non agri income * Self-cultivated land (2000)			−24.9465	−24.7373
			(1.97)	(1.94)
Change in the proportion of non agri income * Years of schooling (labors, 2000)			−8.1823	−8.0409
			(1.35)	(1.33)
Change in migration rate			−38.3717	−49.3338
			(0.36)	(0.45)
Change in migration rate * Self-cultivated land (2000)			49.9934^∗∗^	51.2942^∗∗^
			(2.78)	(2.92)
			−10.8734	−11.0892
			(0.89)	(0.84)
No. of plot	−2.0780	18.0790	−3.2762	14.2783
	(0.27)	(0.94)	(0.41)	(0.86)
No. of plot^2^		−1.9580		−1.7077
		(1.30)		(1.29)
Land owned (2000)	6.9892	7.0269	7.2108	6.9851
	(1.02)	(1.02)	(1.60)	(1.55)
Number of labors (2000)	−9.9131	−10.6000^∗^	−12.1565^∗^	−12.7658^∗^
	(1.78)	(2.08)	(2.08)	(2.36)
Female (labor, 2000)	6.7640	7.1242	5.7277	6.0187
	(0.71)	(0.73)	(0.55)	(0.57)
Age (labor, 2000)	−0.9446	−0.9274	−1.1726	−1.1523
	(0.89)	(0.89)	(1.08)	(1.07)
Years of schooling (labor, 2000)	0.0185	0.2166	2.5423	2.7222
	(0.00)	(0.06)	(0.45)	(0.46)
Net crop income (yuan, 2000)	−0.0018	−0.0018	−0.0014	−0.0015
	(1.15)	(1.18)	(0.82)	(0.86)
Province dummies	Yes	Yes	Yes	Yes
*N*	905	905	905	905
*R*^2^	0.084	0.086	0.103	0.105

*Note:* Absolute *t* statistics in parentheses; ^*^p < 0.10, ^**^p < 0.05, ^***^p < 0.01.

**Table 6:** Robustness check: Village fixed effects

Dependent variable	Self-cultivated land (mu)		Net rent-in land (mu)		Change in machine service (yuan)	
Real wage growth: Non ag * Land owned (2000)	−0.0842	−0.0316	−0.0675	−0.0391	−1.3165	−1.1815
	(0.99)	(0.38)	(1.06)	(0.52)	(0.25)	(0.24)
Real wage growth: Non ag * Years of schooling (labor, 2000)	−0.4138^∗^	−0.4595^∗^	−0.1721	−0.1842	−1.5627	−2.2943
	(1.70)	(1.96)	(1.37)	(1.50)	(0.19)	(0.27)
Real wage growth: Ag * Land owned (2000)	−0.2180	−0.1556	0.0613^∗∗^	0.0670	2.9460^∗^	3.6742
	(1.28)	(1.03)	(2.44)	(1.61)	(1.83)	(1.48)
Real wage growth: Ag * Years of schooling (labors, 2000)	−0.0691	−0.0694	0.0548	0.0503	−0.5937	−0.3163
	(0.80)	(0.73)	(0.65)	(0.56)	(0.13)	(0.07)
Change in the proportion of non agri. income * Land owned (2000)		−0.1799		0.1386		0.0047
		(0.47)		(0.61)		(0.00)
Change in the proportion of non agri. income * Years of schooling (labors, 2000)		−0.3866^∗^		−0.0815		−7.4085
		(1.75)		(0.41)		(0.70)
Change in migration rate * Land owned (2000)		0.9659		0.1917		42.6717
		(1.48)		(0.38)		(1.31)
Change in migration rate * Years of schooling (labors, 2000)		0.4240		0.2393		−33.7187
		(0.73)		(0.51)		(1.25)
Controls included	Yes	Yes	Yes	Yes	Yes	Yes
Village fixed effects	Yes	Yes	Yes	Yes	Yes	Yes
*N*	905	905	905	905	905	905
*R*^2^	0.148	0.153	0.019	0.020	0.014	0.017

*Note:* Absolute *t* statistics in parentheses; ^*^p < 0.10, ^**^p < 0.05, ^***^p < 0.01.

**Table 7:** Crop income

Dependent: Crop income growth (difference in log)	No IV	IV	No IV	IV	No IV	IV	No IV	IV
Sample:	All		All		>6 mu		>6 mu	
Change in land owned	0.1170^∗∗∗^	0.1059^∗∗∗^	0.1408^∗∗∗^	0.1338^∗∗∗^	0.0984^∗∗∗^	0.1143^∗∗∗^	0.1240^∗∗∗^	0.1299^∗∗∗^
	(9.31)	(4.59)	(9.65)	(4.70)	(7.82)	(5.20)	(8.40)	(4.42)
Change in net rent-in land	0.0886^∗∗∗^	0.0933^∗∗∗^	0.0758^∗∗∗^	0.0201	0.0525^∗∗∗^	0.0991^∗∗∗^	0.0192	−0.0062
	(6.20)	(2.98)	(4.52)	(0.49)	(3.69)	(3.72)	(1.14)	(0.17)
Change in mechanic service	−0.0000	−0.0006	−0.0000	−0.0006	−0.0001	0.0005	−0.0002	0.0004
	(0.07)	(1.14)	(0.06)	(1.00)	(0.64)	(0.84)	(0.81)	(0.70)
Change in machine service * change in land owned			−0.0001^∗∗∗^	−0.0001			−0.0001^∗∗^	−0.0000
			(2.79)	(1.31)			(2.10)	(0.28)
Change in machine service * change in net rent-in land			0.0000	0.0002^∗^			0.0001^∗∗^	0.0003^∗∗∗^
			(0.69)	(1.86)			(2.47)	(3.44)
Village fixed effects	Yes	Yes	Yes	Yes	Yes	Yes	Yes	Yes
*N*	605	605	605	605	239	239	239	239
*R*^2^	0.171		0.187		0.255		0.318	
Hausman Test of IV: Chi squared		2.36		5.32		8.91		16.86
*P*-value		0.50		0.38		0.03		0.00

*Note:* Absolute *t* statistics in parentheses; ^*^p < 0.10, ^**^p < 0.05, ^***^p < 0.01.

## Footnotes

1 China’s agriculture is dominated by smallholders with relatively small farm size and fragmented plots. There are about 200 million small farms that produced most of the crops in China (NBSC, 2013). When China completed its household responsibility reform which allocated village land equally to all households in each village in 1985, the average farm size was only 0.7 hectare. Each household normally has at least 3–4 plots of different qualities and some have more than 10 plots. Around 60% of the plots are under 0.1 ha and nearly a quarter are larger than 0.15 ha, with the rest between 0.1 and 0.15 ha (Tan, Heerink, & Qu, 2006).

2 With the expansion of off-farm employment, some farmers are predicted to increase their labor supply to non-agricultural sectors, and/or rent out their land to other farmers (Bowlus & Sicular, 2003).

3 Although China has codified a robust framework for the protection of land rights such as the Land Management Law (1998), the Land Contracting Law (2003) and the Property Law (2007), knowledge and practical implementation of these rights still lag in rural areas. The top-down changes to legal and political structures did not solve China’s continued struggles with unrest resulting from the summary appropriation of land by developers and local officials. Farmers in many areas are still being forced to relocate by local officials, often illegally, and local cadres still retain large amounts of money intended to be distributed to farmers as compensation for any public-interest land seizures.

4 For the mechanical service before harvesting, for example plowing, some of smallholders pay after harvest or selling out agricultural products. This mode of payment is generally to the suppliers of mechanical service who are local residents. However, when the suppliers of mechanical service came from other counties or provinces, the smallholders pay cash as soon as work is done.

5 In some regions, a quota of subsidized machine is distributed like a lottery. Only the one who is lucky enough to get the quota is qualified to buy the certain machines with the subsidy.

6 It should be noted that the sudden decline of the proportion of cultivated area under mechanical plowing is because cultivated area has been recorded to increase dramatically from 94.971 million ha in 1999 to 130.039 million ha in 2000.

7 Potentially this would lead to underestimation of rented-out land since those who stopped farming could rent out their land to other farmers.

8 Since a small number of on-farm hired labors are reported in each village, we expect measurement errors in the variable. We used the county-level average if there is no hired labor case. However, we still think it is important to include agricultural wages due to the fact that agricultural labor demand goes up in a particular season to reflect the demand–supply (im)balance, in contrast with non-agricultural wages.

9 Potentially land rent-out can be under-reported if the attrition rate by migration is high. There were 86 households that could not be interviewed in 2008. The attrition analysis in Table 8 shows that land and labor endowments in 2000 significantly affect the attrition probability but wage growth (and its interactions with the household initial conditions) does not, which confirms that our main findings are not subject to attrition bias. The land and labor endowments are controlled in the outcome equations.

10 Here we do not include input variables such as fertilize use etc. since they are endogenous. Note that fixed effects such as soil quality, etc. are differenced out.

11 Though it is essentially prohibited to sell and buy farm land, the allocation of own land could change over time. However, a large portion of changes in self-cultivated land comes from land rental markets.

12 This could be due to the reverse causation: In areas where cultivation size can easily expand, people do not migrate much.

13 It is possible that rented in land is used for crops different from the main crop in their own land, and the new crops need the use of machines.
